# Distinct effect of preconditioning with p38 MAPK signals on matrix-expanded human synovium-derived stem cell chondrogenesis: sb203580 favors chondrogenic differentiation while anisomycin benefits endochondral bone formation

**DOI:** 10.3389/fcell.2025.1655408

**Published:** 2025-10-23

**Authors:** Ying Zhang, Ming Pei, Chaoliang Lv

**Affiliations:** ^1^ Jinfeng Laboratory, Chongqing, China; ^2^ Department of Spine Surgery, Jining NO.1 People’s Hospital Affiliated to Shandong First Medical University & Shandong Academy of Medical Sciences, Jining, China; ^3^ Stem Cell and Tissue Engineering Laboratory, Department of Orthopaedics, West Virginia University, Morgantown, WV, United States; ^4^ WVU Cancer Institute, Robert C. Byrd Health Sciences Center, West Virginia University, Morgantown, WV, United States

**Keywords:** extracellular matrix, p38 MAPK signal, mesenchymal stem cell, chondrogenic potential, non-canonical wnt signal

## Abstract

**Introduction:**

Cartilage defects are often accompanied by inflammation, presenting a major challenge in clinical treatment. Adult stem cells offer a promising approach for cartilage regeneration; however, *in vitro* expansion leads to replicative senescence, hindering their application. Our previous studies have demonstrated that decellularized extracellular matrix (dECM) can serve as an *in vitro* “microenvironment” to promote stem cell expansion and chondrogenic potential. In this study, we hypothesized that pretreatment with p38 mitogen-activated protein kinase (MAPK), a key pathway driving inflammation, would impair chondrogenesis in dECM-expanded adult stem cells.

**Methods:**

Human synovium-derived stem cells (SDSCs) were expanded for one passage on either dECM or plastic culture flasks and pretreated with p38 MAPK, followed by chondrogenic or osteogenic induction.

**Results:**

We found that pretreatment with sb203580, a p38 MAPK inhibitor, enhanced chondrogenic differentiation of dECM-expanded SDSCs, whereas pretreatment with anisomycin, a p38 MAPK activator, favored both chondrogenic hypertrophy and osteogenic differentiation of dECM-expanded SDSCs. In SDSC pretreatment, p38 MAPK significantly upregulated the non-canonical Wnt signaling pathway during dECM expansion and chondrogenic induction. The significant upregulation of Wnt5a induced by anisomycin combined with dECM expansion may indicate the highest osteogenic potential; SDSC pretreatment with sb203580 combined with dECM expansion exhibited the strongest chondrogenic differentiation and the highest levels of Wnt11.

**Discussion:**

This study suggests that p38 MAPK pretreatment may play a key role in dECM-expanded tissue-specific stem cell-mediated cartilage regeneration. Further verification of Wnt-related regenerative mechanisms remains to be determined.

## Introduction

Cartilage defects do not readily heal spontaneously due to a lack of blood supply. Although adult stem cells are a promising cell source for cartilage regeneration (Toh et al., 2014), *in vitro* expansion, a necessary step prior to *in vivo* application, remains a major challenge for adult stem cells to survive replicative senescence (Li and Pei, 2012). Accumulating evidence suggests that expansion on decellularized extracellular matrix (dECM), a microenvironment resembling an *in vivo* “niche”, is an effective method for rejuvenating adult stem cells in terms of proliferation and chondrogenic potential (Pei, 2017). The effects of dECM preconditioning on tissue-specific stem cell cartilage regeneration have also been demonstrated *in vivo* (Pei et al., 2013a; Pei et al., 2022).

Because post-traumatic joint inflammation is often accompanied by cartilage defects, inflammation may contribute to the reduced efficacy of dECM for adult SDSCs, hindering dECM-mediated cell rejuvenation (Pei et al., 2013a; Yan et al., 2020; Zhang et al., 2015). Fortunately, p38 mitogen-activated protein kinase (MAPK) has been shown to play an important role in stress and inflammatory signals, although it was originally considered a mediator of growth and development (Joos et al., 2009; Long and Loeser, 2010). p38 MAPK has been identified as a major signaling pathway activated by degradative cytokines such as interleukin 1beta (IL-1β) and tumor necrosis factor alpha (TNFα) (Martel-Pelletier et al., 1999; Sondergaard et al., 2010). Furthermore, it has been reported that basal levels of p38 phosphorylation were reduced in osteoarthritic chondrocytes compared with normal chondrocytes, while p38 MAPK inhibitors upregulated the expression of chondrogenic and hypertrophic genes in normal rat chondrocytes (Prasadam et al., 2012).

p38 MAPK inhibitors are used to treat cartilage defects to suppress inflammation; however, the p38 MAPK inhibitor also blocks chondrogenesis (Oh et al., 2000). To exploit their anti-inflammatory effect and limit their inhibitory effects on cartilage regeneration, this study evaluated the potential effects of p38 MAPK inhibitors or activators on chondrogenesis during SDSC pretreatment and cell expansion. Given the interplay between MAPK and Wnt signaling during cartilage regeneration (Zhang et al., 2014), we investigated whether Wnt signaling is also actively involved in the enhanced chondrogenic differentiation of SDSCs following pretreatment with p38 MAPK inhibitors or activators combined with dECM expansion.

## Materials and methods

### SDSC culture

Adult human SDSCs were purchased from Asterand (North America Laboratories, Detroit, MI) (Pei et al., 2013b) and isolated from four donors: two men and two women with a mean age of 43 years and no known joint diseases. These SDSCs were cultured in growth medium consisting of αMEM (alpha minimum essential medium) supplemented with 10% FBS (fetal bovine serum), 100 U/mL penicillin, 100 μg/mL streptomycin, and 0.25 μg/mL fungizone (Invitrogen, Carlsbad, CA). Cultures were maintained in a humidified incubator at 37 °C with 5% CO_2_, and the growth medium was changed every 3 days.

### dECM preparation

The preparation of dECM has been described previously (Li and Pei, 2018). Briefly, Plastic (tissue culture plastic) was pre-coated with 0.2% gelatin (MilliporeSigma, Burlington, MA) and incubated at 37 °C for 1 h, followed by fixation with 1% glutaraldehyde and treatment with 1M ethanolamine. P3 (passage 3) SDSCs were seeded at a density of 6,000 cells/cm^2^ and cultured to 90% confluence. Growth medium was supplemented with 250 μM L-ascorbic acid phosphate (Wako Chemicals United States, Inc., Richmond, VA) and cultured for 8 days. The deposited matrix was treated with 0.5% Triton X-100 containing 20 mM ammonium hydroxide at 37 °C for 5 min to remove cells and then stored in PBS (phosphate-buffered saline) containing 100 U/mL penicillin, 100 μg/mL streptomycin, and 0.25 μg/mL fungizone at 4 °C.

### Morphological characterization of dECM with or without SDSCs

After post-fixation in 2.5% glutaraldehyde (MilliporeSigma) for 2 hrs, representative samples (n = 2) were re-post-fixed in 2% osmium tetroxide (MilliporeSigma) for 2 hrs. Samples were dehydrated using a graded ethanol series and then treated twice with HMDS (hexamethyldisilazane) (MilliporeSigma) mixed with ethanol at a 1:1 ratio for 1 hour each, followed by overnight treatment with HMDS at a 1:2 ratio with ethanol, and finally three times with HMDS for 4 hrs each. After air-drying for 24-h, samples were gold-sputtered. Images were captured using a scanning electron microscope (SEM) (Hitachi, Model S 2400).

### Global gene expression by microarrays and data analyses

P3 SDSCs were expanded for one passage on dECM or Plastic and then induced into chondrogenic tissue for 21 days in a pellet culture system (designated as Ep and Pp, respectively). Total RNA was purified from the expanded cells and 21-day pellets using TRIzol (Invitrogen) and further purified using the RNeasy Mini Kit (Qiagen, Valencia, CA) according to the manufacturer’s instructions. The specified amount of cDNA (5.5 µg) was then fragmented and biotinylated using the GeneChip® WT Terminal Labeling Kit (Affymetrix, Santa Clara, CA). This entire reaction mixture, containing 50 µL of fragmented biotinylated cDNA and hybridization controls, was hybridized to a Human GeneChip® 1.0 ST Exon Arrays (Affymetrix) in a GeneChip® 640 Hybridization Chamber (Affymetrix) at 45 °C for 17 h. Parameters such as the scale factor, background noise, and percent presence were calculated according to the manufacturer’s instructions (Affymetrix). The resulting raw data were then uploaded to GeneSpring (Agilent, Santa Clara, CA) and Partek (St. Louis, MO) software for preliminary analysis. Pathway and functional analysis was performed using Ingenuity Pathway Analysis (IPA, Redwood City, CA). Briefly, raw intensity values were adjusted by background subtraction, then normalized by robust multiarray analysis (RMA), log-transformed, and fold-changes assessed. All batch effects related to scan date were eliminated before fold-change calculation.

### SDSC expansion and p38 MAPK pretreatment

P3 SDSCs were seeded at a density of 3,000 cells/cm^2^ on two substrates (dECM and Plastic) for one passage. To investigate the effects of p38 MAPK on the expanded cells, 10 µM sb203580 (a p38 MAPK inhibitor, LC Laboratories, Woburn, MA) or 1 µM anisomycin (a p38 MAPK activator, also from LC Laboratories) was added 48 h after seeding and maintained throughout the culture period. Groups not treated with p38 MAPK served as controls. The experimental design included six groups: dECM expansion group (denoted as Econ), dECM expansion combined with sb203580 group (denoted as Esb), dECM expansion combined with anisomycin group (denoted as Ean), Plastic expansion alone (Pcon), Plastic expansion combined with sb203580 group (denoted as Psb), and Plastic expansion combined with anisomycin group (denoted as Pan). Cell counts were performed using a hemocytometer to determine the cell number in each group.

### Chondrogenic induction of expanded SDSCs

0.3 × 10^6^ SDSCs from each group were placed in a 15-mL polypropylene tube and centrifuged at 500 *g* for 5 min to form a pellet. The pellet was incubated overnight in growth medium and then cultured in serum-free chondrogenic medium for 35 days. This medium consisted of high-glucose DMEM (Dulbecco’s Modified Eagle’s Medium) supplemented with 40 μg/mL proline, 100 nM dexamethasone, 100 U/mL penicillin, 100 μg/mL streptomycin, 0.1 mM ascorbic acid-2-phosphate, and 1×ITS™ Premix (BD Biosciences, San Jose, CA). In addition, 10 ng/mL TGF-β3 (transforming growth factor beta 3) (PeproTech Inc., Rocky Hill, NJ) was added. Chondrogenic differentiation was assessed at 14 and 35 days by histology, immunostaining, biochemical analysis, and real-time qPCR (quantitative polymerase chain reaction).

For histological analysis, representative pellets (n = 3) were fixed with 4% paraformaldehyde overnight at 4 °C and then dehydrated using a graded ethanol series. Samples were cleared with xylene and embedded in paraffin blocks. Histochemical staining of 5-µm-thick sections was performed using Alcian blue (MilliporeSigma) and counterstained with Fast Red to visualize sulfated glycosaminoglycan (sGAG). For immunohistochemistry (IHC) analysis, sections were labeled with primary antibodies against type II collagen (II-II6B3; Developmental Studies Hybridoma Bank, Iowa City, IA), type I collagen (GeneTex Inc., Irvine, CA), type X collagen (MilliporeSigma), and matrix metalloproteinase 13 (MMP13) (VIIIA2, Abcam, Cambridge, MA). A biotinylated horse anti-mouse IgG secondary antibody (Vector, Burlingame, CA) was then applied, and immunoactivity was visualized using Vectastain ABC reagent (Vector) with 3,3′-diaminobenzidine as a substrate.

For biochemical quantification, another representative set of pellets (n = 4) was digested in PBE buffer (100 mM phosphate and 10 mM ethylenediaminetetraacetic acid, pH 6.5) containing 125 μg/mL papain and 10 mM cysteine for 4 hours at 60 °C, using 200 μL of enzyme per sample. DNA concentration in the papain digests was quantified using the Quant-iT™ PicoGreen™ dsDNA Assay kit (Invitrogen) using a CytoFluor® 4000 Series (Applied Biosystems, Foster City, CA). GAG levels were measured using a Spectronic BioMate 3 Spectrophotometer (ThermoFisher Scientific, Milford, MA) using dimethylmethylene blue dye and bovine chondroitin sulfate as a standard.

For qPCR analysis, total RNA from another set of pellets (n = 4) was extracted in TRIzol® (Invitrogen) using an RNase-free pestle. Approximately 1 µg of RNA was reverse transcribed using the High Capacity cDNA Archive Kit (Applied Biosystems) at 37 °C for 120 min. Custom genes included chondrogenic marker genes such as *COL2A1* (type II collagen; assay ID: Hs00156568_m1), *ACAN* (aggrecan; assay ID: Hs00153935_m1), and *SOX9* [SRY (sex determining region Y)-box 9; assay ID: Hs00165814_m1), as well as hypertrophic marker genes *COL10A1* (type X collagen; assay ID: H200166657_m1) and *MMP13* (assay ID: Hs00233992_m1). The endogenous control gene was eukaryotic *18S RNA* (assay ID: Hs99999901_s1). Real-time qPCR was performed using the iCycler iQ™ Multicolor Real-Time PCR Detection System (Perkin-Elmer, Waltham, MA). Relative transcript levels were calculated using the formula χ = 2^−ΔΔCt^, where ΔΔCt = ΔE-ΔC, ΔE = Ct_exp_-Ct_18s_, and ΔC = Ct_ct1_-Ct_18s_.

### Western blot

To investigate the potential effects of dECM expansion combined with p38 MAPK on cell proliferation and chondrogenic differentiation through Wnt signaling, we homogenized expanded cells and chondrogenically differentiated pellets from each group. These samples were dissolved in lysis buffer supplemented with protease inhibitors (Cell Signaling, Danvers, MA). Total protein concentration was then measured using a BCA™ Protein Assay Kit (ThermoFisher Scientific).

For subsequent analysis, 30 μg of protein from each sample was denatured, separated by gel electrophoresis, and transferred to a nitrocellulose membrane (Invitrogen). The membrane was incubated with primary monoclonal antibodies diluted in 5% bovine serum albumin, 1× TBS (10 mM Tris-HCl, 150 mM NaCl, pH 7.5), and 0.05% Tween-20 for 1 h at room temperature. A horseradish peroxidase-conjugated goat anti-mouse secondary antibody (ThermoFisher Scientific) was then added and incubated for 1 h. Blots were exposed using SuperSignal West Femto Maximum Sensitivity Substrate and CL-X exposure film (ThermoFisher Scientific). Primary antibodies used in immunoblotting included Wnt3a, Wnt5a, and Wnt11 (ThermoFisher Scientific) and β-actin (Cell Signaling).

### Osteogenic induction of expanded SDSCs

Expanded SDSCs (n = 3) were reseeded at a density of 8,000 cells per cm^2^. When the cultures reached 90% confluence, the medium was changed to an osteogenic induction medium, consisting of growth medium supplemented with 0.01 μM dexamethasone, 10 mM β-glycerophosphate, 50 μM ascorbate-2-phosphate, and 0.01 μM 1,25-dihydroxyvitamin D3. Induction culture continued for 21 days.

To assess osteogenic differentiation, ALP (alkaline phosphatase) activity was measured using a kit (MilliporeSigma). To assess calcium deposition, induced cells (n = 3) were fixed with 70% ice-cold ethanol for 1 h and then incubated in 40 mM Alizarin Red S (ARS), pH 4.2, for 20 min with agitation. After rinsing twice with deionized water, matrix mineral-binding staining was documented using a Nikon TE300 phase-contrast microscope (Nikon, Japan). Total ALP and calcium accumulation was analyzed using NIH ImageJ software (U.S. National Institutes of Health, Bethesda, MD).

### Statistics

Numerical data are presented as the mean and standard error. Pairwise comparisons in biochemical and real-time qPCR data analysis were performed using the Mann-Whitney U test. All statistical analyses were performed using SPSS 13.0 statistical software (SPSS Inc., Chicago, IL). A *p*-value of less than 0.05 was considered statistically significant.

## Results

### dECM-expanded SDSCs and chondrogenic potential

**FIGURE 1:**
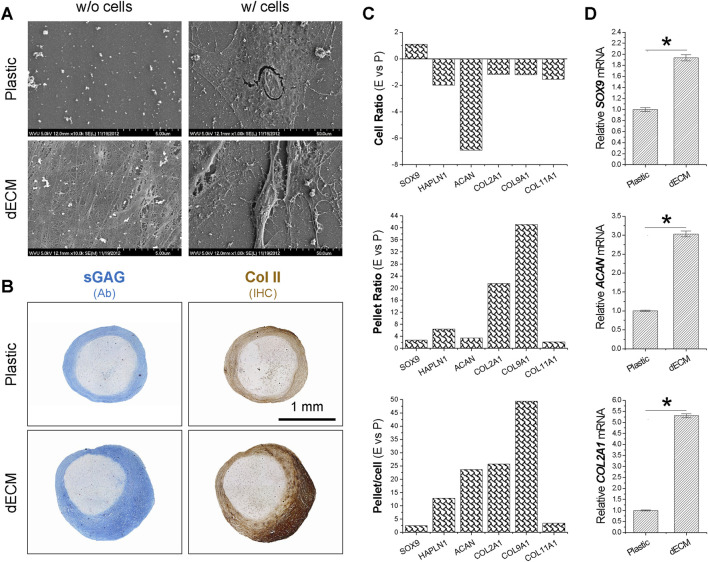
Effects of dECM pretreatment on the chondrogenic potential of SDSCs. Human SDSCs were expanded for one passage on dECM or Plastic and then induced to form chondrocytes. **(A)** SEM was used to evaluate culture substrates (dECM versus Plastic) (w/o cells) and SDSCs expanded on culture substrates (w/cells). **(B)** Alcian blue (Ab) was used to stain sulfated GAG (sGAG) and immunohistochemistry (IHC) was for staining of type II collagen (Col II). Scale bar = 1 mm. **(C)** Microarray was used to measure fold changes in cartilage marker genes during cell expansion (cell ratio) and chondrogenic differentiation (pellet ratio) in SDSCs expanded on dECM versus Plastic (E vs*.* P). **(D)** Real-time qPCR was used to confirm changes in representative cartilage marker genes in SDSCs induced by chondrogenic medium after expansion on dECM. Data are shown as mean ± SD (standard deviation) for n = 4. *p < 0.05 indicates statistical significance.

SEM data (Figure 1A) showed that SDSCs expanded on dECM had a small, fibroblast-like shape, while those grown on Plastic had a flat, broad shape. To determine whether SDSCs expanded on dECM possessed enhanced chondrogenic potential, we induced chondrogenesis in a pellet culture system. Histology data (Figure 1B) revealed that pellets of SDSCs expanded on dECM were not only larger in size but also stained more intensely for sGAG and type II collagen, two typical cartilage markers. Microarray data (Figure 1C) showed that compared to Plastic-expanded cells, dECM-expanded SDSCs showed upregulation of chondrogenic markers [*HAPLN1* (hyaluronan and proteoglycan link protein 1), *ACAN*, *COL2A1*, *COL9A1* (type IX collagen), and *COL11A1* (type XI collagen)]. Notably, dECM-expanded chondrogenically induced SDSCs exhibited upregulation of these chondrogenic markers, consistent with histology data (Figure 1B). Interestingly, the chondrogenic transcription factor SOX9 remained upregulated during both the dECM expansion period and the subsequent chondrogenic induction period (Figure 1C). These microarray data were also confirmed by real-time qPCR analysis of *SOX9*, *ACAN*, and *COL2A1* expression in chondrogenically induced SDSCs (Figure 1D).

### Effect of dECM pretreatment on wnt signaling during sdsc expansion and subsequent chondrogenic induction

**FIGURE 2:**
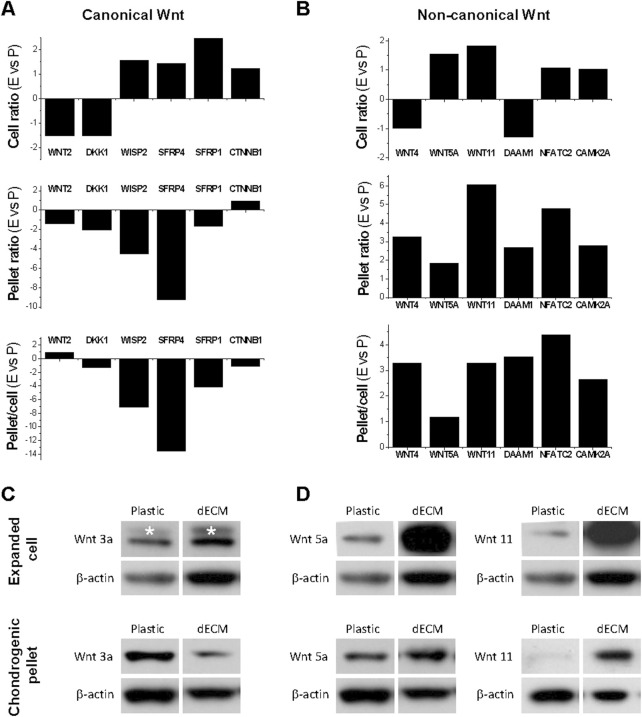
Effects of dECM pretreatment on Wnt signaling changes after SDSC expansion and subsequent chondrogenic induction. Human SDSCs were expanded on dECM or Plastic for one passage and then subjected to chondrogenic induction. Microarray was used to measure the fold changes in canonical **(A)** and non-canonical **(B)** Wnt signaling-related genes during cell expansion (cell ratio) and chondrogenic differentiation (pellet ratio) in SDSCs expanded on dECM versus Plastic (E vs*.* P). Western blot was used to confirm the above changes in canonical **(C)** and non-canonical **(D)** Wnt signaling in chondrogenically induced SDSCs after expansion on dECM. β-actin was used as a loading control.

To determine the involvement of Wnt signaling in dECM expansion, we used microarray to analyze canonical and non-canonical Wnt signaling in SDSCs expanded on dECM and Plastic, and during subsequent chondrogenic induction. In Figure 2A, SDSCs expanded on dECM showed slight downregulation of the canonical Wnt signaling antagonist *DKK1* [dickkopf 1 homolog (*Xenopus laevis*)] and slight upregulation of the Wnt signaling pathway activator *CTNNB1* (cadherin-associated protein beta 1), suggesting that canonical Wnt signaling may be enhanced during dECM-mediated cell expansion. However, microarray data did not detect a fold change in *WNT3A* (a typical canonical Wnt signaling ligand) after SDSC expansion on dECM (but not on Plastic), despite a dramatic decrease in dECM-expanded chondrogenically induced SDSCs, as confirmed by Western blotting data (Figure 2B). Notably, dECM expansion upregulated *WNT5A* and *WNT11*, as well as *NFATC2* (nuclear factor of activated T-cells, cytoplasmic, calcineurin-dependent 2) and *CAMK2A* (calcium/calmodulin-dependent protein kinase type II alpha chain) in expanded and chondrogenically induced SDSCs (Figure 2C). This finding indicates that non-canonical Wnt signaling may be involved in dECM-mediated SDSC rejuvenation in terms of cell proliferation and chondrogenic potential, as confirmed by Western blotting data (Figure 2D).

### Pretreatment with p38 MAPK inhibitor enhanced the chondrogenic potential of SDSCs, while p38 MAPK activator promoted chondrogenic hypertrophy, particularly in SDSCs expanded on dECM

**FIGURE 3:**
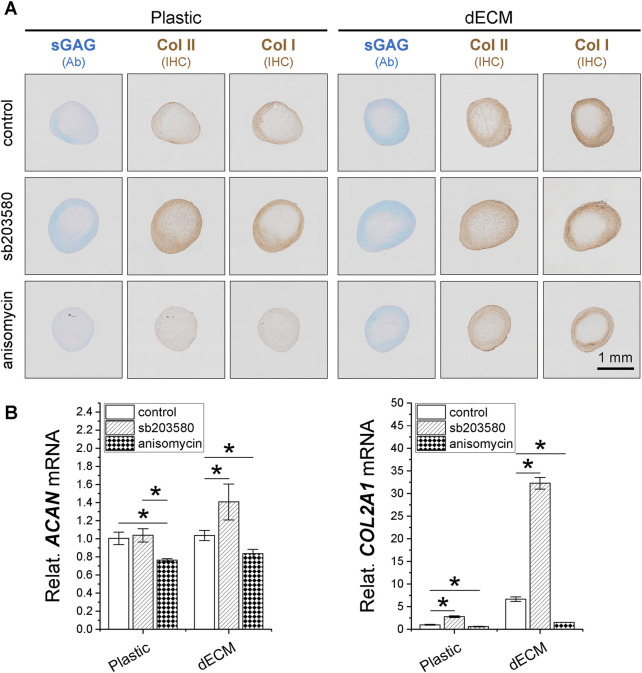
Effects of SDSC pretreatment [p38 MAPK inhibitor (sb203580) or activator (anisomycin)] combined with dECM expansion on their chondrogenic potential. Human SDSCs were expanded for one passage on dECM or Plastic in the presence of sb203580 or anisomycin, followed by chondrogenic induction for 14 and 35 days. **(A)** Alcian blue (Ab) was used to stain sGAG, and immunohistochemistry (IHC) was for staining of type I collagen (Col I) and type II collagen (Col II). Scale bar = 1 mm. **(B)** Real-time qPCR was used for *ACAN* and *COL2A1.* Data are shown as mean ± SD for n = 4. *p < 0.05 indicates statistical significance.

To determine whether pretreatment with p38 MAPK inhibitor plays a role in SDSC expansion and chondrogenic potential, 10 µM sb203580 was added to the culture medium of SDSCs expanded on Plastic or dECM; 1 µM anisomycin (an activator of p38 MAPK) was also added as a control. Although dECM expansion increased cell number by 1.19-fold compared to Plastic expansion, supplementation with sb203580 increased cell number both when expanded on dECM (1.21-fold versus 0.20-fold) and Plastic (1.85-fold versus 0.44-fold), whereas anisomycin reduced this increase. Surprisingly, sb203580 pretreatment also increased the size of chondrogenically SDSC pellets on day 14, with comparable staining intensity for sGAGs and type II collagen; the benefits of sb203580 pretreatment on the chondrogenic potential of dECM-expanded SDSCs were amplified (Figure 3A). These histological data were supported by real-time qPCR data (Figure 3B).

**FIGURE 4:**
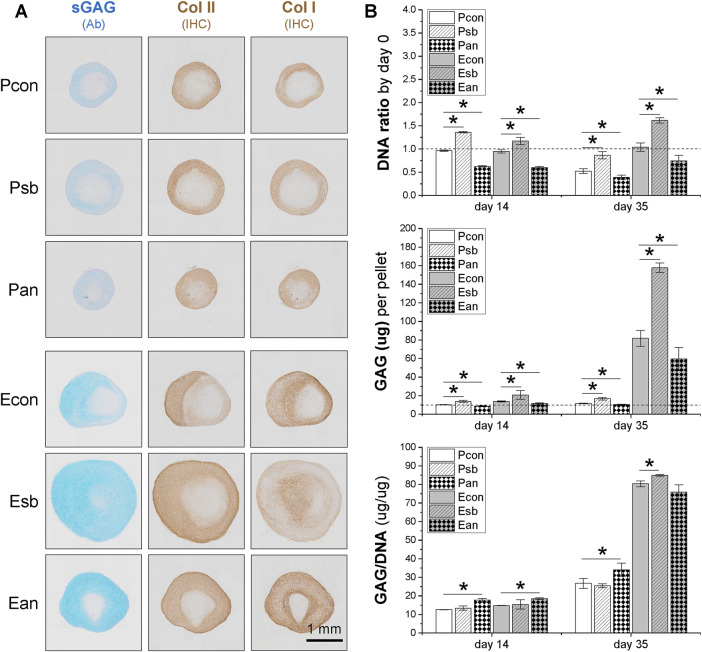
Effects of SDSC pretreatment [p38 MAPK inhibitor (sb203580) or activator (anisomycin)] combined with dECM expansion on SDSC chondrogenic potential. Human SDSCs were expanded for one passage on dECM or Plastic in the presence of sb203580 or anisomycin, followed by 35 days of chondrogenic induction. **(A)** Alcian blue (Ab) was used to stain sGAG, and immunohistochemistry (IHC) was for staining of type I collagen (Col I) and type II collagen (Col II). Scale bar = 1 mm. **(B)** Biochemical analysis was used to analyze the DNA and GAG contents in the chondrogenic pellets. Cell proliferation and viability were assessed using the DNA ratio (DNA content on days 35 and 14 were adjusted to the DNA content on day 0). The chondrogenic index was assessed using the GAG to DNA ratio. Data are shown as mean ± SD for n = 4. *p < 0.05 indicates statistical significance.

The 35-day pellets further confirmed the data from the 14-day pellets described above, with dECM-expanded SDSCs producing larger pellets (Figure 4A) and higher cell viability and GAG content compared to the corresponding Plastic group (Figure 4B). Pretreatment with dECM and sb203580 produced the largest SDSC pellets (Figure 4A) with the highest cell viability and GAG content, followed by the dECM group, while the anisomycin-combination group had the least viability (Figure 4B). In contrast, pretreatment with anisomycin slightly reduced the pellet size of chondrogenically differentiated SDSCs, despite comparable staining intensity for sGAG and type II collagen (Figure 4A); cell viability and GAG content did not decrease significantly (Figure 4B).

**FIGURE 5:**
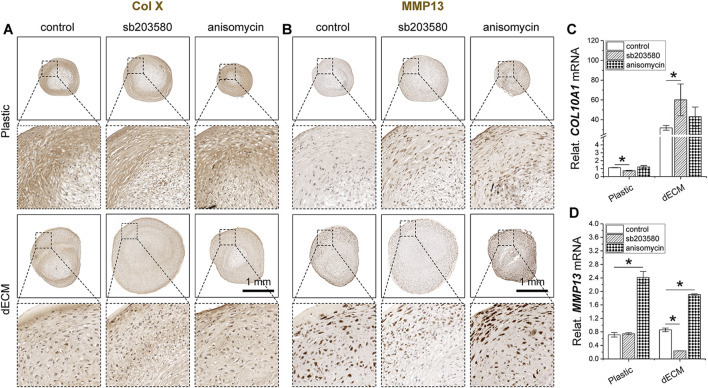
Effects of SDSC pretreatment [p38 MAPK inhibitor (sb203580) or activator (anisomycin)] combined with dECM expansion on SDSC chondrogenic hypertrophy. Human SDSCs were expanded for one passage on dECM or Plastic in the presence of sb203580 or anisomycin, followed by 35 days of chondrogenic induction. Immunohistochemistry (IHC) staining of type X collagen (Col X) **(A)** and MMP13 **(B)** in pellets was performed. Scale bar = 1 mm. Real-time qPCR for *COL10A1* mRNA **(C)** and *MMP13* mRNA **(D)** was performed to validate the histological staining. Data are shown as mean ± SD for n = 4. *p < 0.05 indicates a statistically significant difference.

Intriguingly, sb203580 pretreatment reduced the intensity of type X collagen immunostaining in SDSC pellets in the Plastic group, whereas no significant difference was observed in the dECM group (Figure 5A), consistent with the real-time qPCR data (Figure 5C). Anisomycin pretreatment enhanced immunostaining of MMP13, another hypertrophic marker, in SDSC pellets in both the Plastic and dECM groups, while sb203580 pretreatment resulted in the weakest MMP13 immunostaining in SDSCs in the dECM group (Figure 5B), which was supported by real-time qPCR data (Figure 5D).

### WNT5A was upregulated in chondrogenically differentiated SDSCs pretreated with p38 MAPK activator, while WNT11 was upregulated in chondrogenically differentiated SDSCs pretreated with p38 MAPK inhibitor

**FIGURE 6:**
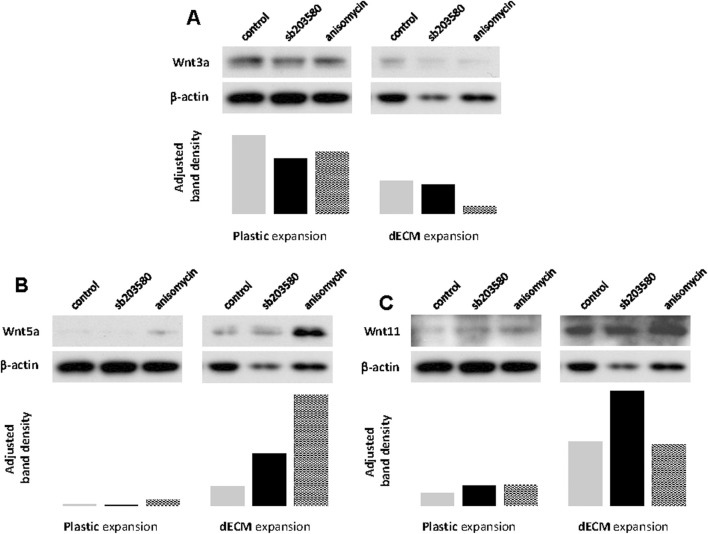
Involvement of non-canonical Wnt signaling during pretreatment with dECM and p38 MAPK signaling. Human SDSCs expanded on dECM or Plastic in the presence of sb203580 or anisomycin were evaluated for canonical **(A)** Wnt3a) and non-canonical **(B)** Wnt5a and **(C)** Wnt11) signaling. β-actin served as a loading control. Immunoblotting bands were semi-quantitatively analyzed using ImageJ software.

To determine whether the Wnt pathway is involved in the chondrogenic potential of SDSCs expanded on dECM pretreated with p38 MAPK, we assessed canonical and non-canonical Wnt signaling in chondrogenically differentiated SDSCs using Western blotting. We found that Wnt3a expression was upregulated in chondrogenically differentiated SDSCs expanded on dECM compared to Plastic (Figure 6A). We also found that both Wnt5a (Figure 6B) and Wnt11 (Figure 6C) expression was upregulated in chondrogenically differentiated SDSCs expanded on dECM compared to Plastic. Notably, Wnt5a expression levels were highest in SDSC pellets generated when dECM expansion was combined with anisomycin (Figure 6B), while Wnt11 expression levels were highest in SDSC pellets generated when dECM expansion was combined with sb203580 (Figure 6C).

### Pretreatment with a p38 MAPK activator enhanced the osteogenic potential of dECM-Expanded SDSCs

**FIGURE 7:**
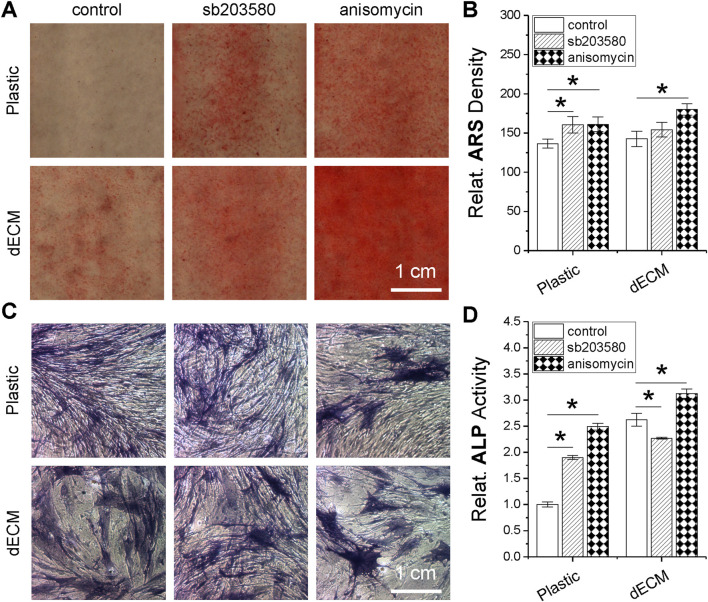
Effects of SDSC pretreatment [p38 MAPK inhibitor (sb203580) or activator (anisomycin)] combined with dECM expansion on SDSC osteogenic potential. Human SDSCs were expanded for one passage on dECM or Plastic in the presence of sb203580 or anisomycin, followed by 21 days of osteogenic induction. Alizarin Red S (ARS) was used for calcium deposition staining **(A)** and staining density was quantified **(B)**. Alkaline phosphatase (ALP) staining was also performed **(C)**, and activity was quantified **(D)**. Data are shown as mean ± SD for n = 4. *p < 0.05 indicates statistical significance.

To further determine whether pretreatment with a p38 MAPK activator plays a role in the osteogenic potential of SDSCs, dECM- or Plastic-expanded cells (with or without pretreatment of sb203580 and anisomycin) were incubated in osteogenic induction medium for 21 days. Our ARS staining revealed that supplementation with either sb203580 or anisomycin enhanced calcium deposition in expanded cells, although no significant differences were observed between dECM and Plastic expansion. Interestingly, combined dECM expansion and anisomycin pretreatment resulted in the highest staining intensity (Figure 7A), which was confirmed by ARS quantification data (Figure 7B). ALP staining (Figure 7C) and activity analysis (Figure 7D) also demonstrated that combined dECM expansion and anisomycin pretreatment resulted in expanded SDSCs with the highest ALP activity, another marker of osteogenic differentiation.

## Discussion

Despite the increasing application of dECM in cell-based cartilage regeneration (Pei et al., 2013b; Pei et al., 2022), little is known about the involvement of the MAPK and Wnt signaling pathways in the proliferation of dECM-expanded cells and their subsequent chondrogenic differentiation. Our previous reports have shown that non-canonical Wnt signaling is upregulated in dECM-mediated expansion and chondrogenic induction of SDSCs (Li et al., 2014; Li et al., 2020). To further investigate whether pretreatment with p38 MAPK affects the chondrogenic differentiation capacity of expanded cells and the associated changes in Wnt signaling, in this study, we expanded SDSCs for one passage on dECM or Plastic in the presence of sb203580 or anisomycin. We found that sb203580 pretreatment favored chondrogenic differentiation of dECM-expanded SDSCs, whereas anisomycin pretreatment favored chondrogenic hypertrophy of dECM-expanded SDSCs. Osteogenic induction studies also demonstrated the contribution of anisomycin to endochondral bone formation of dECM-expanded SDSCs. During dECM expansion and chondrogenic differentiation, the non-canonical Wnt signaling pathway was greatly upregulated in SDSCs pretreated with p38 MAPK. In SDSCs pretreated with anisomycin combined with dECM expansion, significant upregulation of Wnt5a was associated with the highest osteogenic potential whereas, in SDSCs pretreated with both sb203580 and dECM expansion, the highest chondrogenic differentiation was associated with the highest levels of Wnt11.

Dedifferentiation refers to a cellular process in which differentiated cells revert to an earlier developmental stage and may be involved in regeneration (Casimir et al., 1988). Alternatively, cells cultured in a monolayer may lose their original properties, such as protein expression, or change shape. For instance, proliferation and differentiation are unique features of human chondrocytes; *in vitro* expansion often results in a loss of differentiation (Schnabel et al., 2002). Similar to the former definition, dECM expansion can lead to “dedifferentiation”, resulting in expanded SDSCs possessing enhanced chondrogenic potential. This process is distinct from the “dedifferentiation” induced by monolayer culture, in which dedifferentiated chondrocytes typically irreversibly lose their chondrogenic potential (Li and Pei, 2012). Downregulation of chondrogenic differentiation genes (such as *ACAN*) and upregulation of chondrogenic transcriptional genes (such as *SOX9*) during dECM expansion may be one of the mechanisms by which mesenchymal stem cells (MSCs) maintain stemness and chondrogenic potential (Wang et al., 2021).

Research into the mechanisms underlying dECM expansion and its rejuvenation ability on stem cells is in its infancy and warrants further investigation. Wnt signaling plays a particularly important role in regulating MSC proliferation and differentiation. Our microarray data suggest that the Wnt pathway is involved in SDSC dECM expansion and subsequent chondrogenic differentiation. Canonical Wnt signaling, mediated by Wnt3a, has been reported to maintain stem cells in an undifferentiated and highly proliferative state (Kawakami et al., 2001; Reya et al., 2003). However, we found that, regardless of whether SDSCs were expanded on dECM or Plastic, dECM expansion resulted in a higher proliferation rate, but upregulation of Wnt3a mRNA (from microarray) or protein levels (from Western blot) was not significant. Intriguingly, after chondrogenic induction, dECM-expanded SDSCs exhibited a rapid upregulation of Wnt3a, which may be due in part to the inhibition of chondrogenesis by Wnt3a, leading to accelerated chondrogenic differentiation (Reinhold et al., 2006). While all Wnt inhibitors were upregulated during chondrogenesis, we also found that *WISP2* (WNT1-inducible-signaling pathway protein 2), *SFRP1* (Secreted frizzled-related protein 1), and *SFRP4* were also upregulated during the proliferation phase, when *DKK1* was upregulated, suggesting a delicate balance between canonical Wnt signaling in dECM-expanded SDSCs. Compared to the relatively stable canonical Wnt signaling, non-canonical Wnt signaling (such as *WNT5A* and *WNT11* and their downstream effectors *NFATC2* and *CAMK2A*) was significantly upregulated in dECM-expanded SDSCs during both the proliferation and chondrogenesis phases, as confirmed by our Western blot data at the protein level. It is well known that non-canonical signaling can antagonize canonical Wnt activity (Maye et al., 2004; Topol et al., 2003). Our results indicate that non-canonical Wnt signaling increases with dECM expansion until a balance is reached between canonical and non-canonical Wnt signaling. However, during chondrogenic induction, this balance was disrupted, with canonical Wnt signaling downregulated and non-canonical Wnt signaling upregulated in dECM-expanded SDSCs.

dECM expansion promoted cell proliferation, and concurrently upregulated Wnt5a levels, suggesting a possible parallel between cell proliferation and Wnt5a levels. Wnt5a-mediated non-canonical Wnt signaling has been reported to regulate endothelial cell proliferation (Cheng et al., 2008), promote fibroblast proliferation, and enhance relative resistance to hydrogen peroxide induced apoptosis (Vuga et al., 2009). Our data corroborated these findings, with dECM expansion leading to significant upregulation of Wnt5a and Wnt11, but not Wnt3a. The primary function of Wnt5a during early chondrogenesis is to cooperate with other Wnt antagonists to maintain low levels of canonical signaling, thereby enabling cartilage differentiation (Hosseini-Farahabadi et al., 2013). Studies have shown that supplementation of Wnt5a during chondrogenesis promotes chondrogenic differentiation but inhibits hypertrophic chondrocyte differentiation (Bradley and Drissi, 2010). Wnt5a also reduces cell-cell adhesion (Torres et al., 1996), consistent with the fact that cell-matrix interactions govern dECM expansion and are responsible for active migration (Lin et al., 2012). Our results also suggest the possibility of a canonical WNT-to-WNT11 signaling loop, in which canonical WNT signaling induces *WNT11* upregulation, which then activates a non-canonical WNT signaling cascade to induce cellular motility, as recently described (Katoh and Katoh, 2009). *WNT11* can also activate the Ca^2+^-MAP3K7-NLK signaling cascade, thereby attenuating canonical WNT signaling (Katoh and Katoh, 2009). Although Wnt11 was upregulated in dECM-expanded SDSCs, current evidence is insufficient to support a self-renewal function for Wnt11 (Singla et al., 2006) which needs to be further elucidated in future studies.

The p38 MAPK signaling cascade is not only involved in cell proliferation and differentiation (Onom and Han, 2000) but is also activated by various pro-inflammatory and stressful stimuli (Schindler et al., 2007). Evidence suggests that *in vivo* administration of p38 MAPK inhibitor can alleviate inflammation and associated damage; however, these inhibitors can also have side effects, such as the suppression of tissue regeneration (Yong et al., 2009). Interestingly, in this study, we found that preconditioning stem cells with p38 MAPK inhibitor during expansion significantly enhanced dECM-mediated chondrogenesis in SDSCs, likely by preventing inflammation. We also found that pretreatment with sb203580 promoted chondrogenic differentiation of dECM-expanded SDSCs, concomitantly with upregulation of Wnt11 expression, suggesting that p38 MAPK inhibitors may enhance Wnt11-mediated chondrogenesis in dECM-expanded SDSCs. Although Wnt11 plays multiple roles in regulating cell properties (Ouko et al., 2004; Lako et al., 1998; Sekiya et al., 2002), its precise functions and mechanisms of action remain unclear. Unlike the canonical Wnts, Wnt11 is classified as one of the few recognized pro-differentiation Wnts, acting through a β-catenin-independent pathway involving PKC (protein kinase C) and Jnk (c-Jun N-terminal kinase) (Eisenberg et al., 1997; Pandur et al., 2002).

We also found that pretreatment with anisomycin promoted osteogenic differentiation of dECM-expanded SDSCs and upregulated Wnt5a expression, suggesting that anisomycin may enhance Wnt5a-mediated osteogenesis in dECM-expanded SDSCs. This finding is consistent with numerous reports. For example, Lee et al. found that osteogenic transcription factor Runx2 (Runt-related transcription factor-2) was effectively inhibited by sb203580, while anisomycin significantly induced its expression (Lee et al., 2002); Wnt5a plays a key role in the osteogenic differentiation of human MSCs both *in vitro* and *in vivo* (Guo et al., 2008; Baksh and Tuan, 2007). Given that Wnt5a signaling leads to intracellular Ca^2+^ release and activation of PKC and CaMKII (calcium/calmodulin-dependent protein kinase II) (Kühl et al., 2000), upregulation of Wnt5a through pretreatment with anisomycin may predispose SDSCs to osteogenic development. Our assessment of SDSC chondrogenic hypertrophy supports this conclusion. In this study, pretreatment with sb203580 reduced early hypertrophic markers (such as *COL10A1*), whereas anisomycin increased them in expanded SDSCs. Our hypertrophy analysis was performed on 35-day chondrogenically induced SDSC pellets, which exceeds the chondrogenic hypertrophy phase of human MSCs (typically occurring between 14 and 28 days after incubation in a TGF-β-chondrogenic medium) (Mueller et al., 2010). This stage of endochondral ossification is regulated by MMPs (Ortega et al., 2004). Our data showed that pretreatment with anisomycin significantly upregulated *MMP13* in chondrogenically differentiated SDSCs, whereas pretreatment with sb203580 upregulated *MMP13* in dECM-expanded SDSCs, suggesting that pretreatment with p38 MAPK activator favors endochondral ossification, while pretreatment with p38 MAPK inhibitor favors chondrogenic differentiation. Furthermore, secreted MMP13 can degrade type II collagen (Inada et al., 2004) and aggrecan (Fosang et al., 1996), which may explain why pretreatment with p38 MAPK inhibitor resulted in more functional cartilage matrix, whereas pretreatment with p38 MAPK activator produced less functional cartilage matrix.

## Conclusion

**FIGURE 8:**
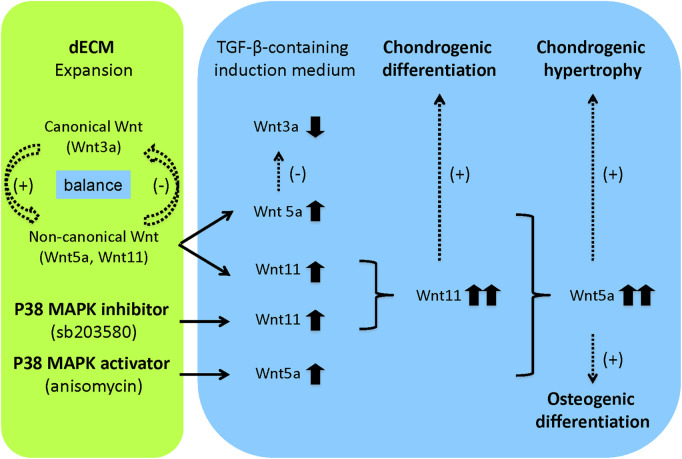
Flowchart of future research hypotheses. In this study, the dECM model provided a cell expansion matrix to maintain a balance of canonical and non-canonical Wnt signaling, thereby promoting expanded cell proliferation, migration, and chondrogenic potential. When expanded cells were released from the dECM and induced to chondrogenesis in a defined medium containing TGF-β, a shift from canonical to non-canonical Wnt signaling occurred, favoring chondrogenic differentiation. Pretreatment with a p38 MAPK inhibitor confirmed the effect of dECM on the chondrogenic potential of expanded SDSCs, possibly through interaction with Wnt11-mediated signaling. Pretreatment with a p38 MAPK activator accelerated the effect of dECM on the osteogenic potential of expanded SDSCs, perhaps through interaction with Wnt5a-mediated signaling. Dotted arrows indicated hypotheses that require verification in future studies.

Our results suggest that pretreatment with p38 MAPK signaling plays distinct roles in matrix-expanded SDSC chondrogenesis: sb203580 favors chondrogenic differentiation, while anisomycin favors endochondral bone formation. Our Wnt signaling data also suggest a novel hypothesis (Figure 8): pretreatment with a p38 MAPK inhibitor may underlie the effects of dECM on the chondrogenic potential of expanded SDSCs through interaction with Wnt11-mediated signaling, whereas pretreatment with a p38 MAPK activator may accelerate the effects of dECM on the osteogenic potential of expanded SDSCs through interaction with Wnt5a-mediated signaling. This hypothesis warrants further investigation.

## Data Availability

The datasets presented in this study can be found in online repositories. The names of the repository/repositories and accession number(s) can be found in the article/supplementary material.
